# IgA Nephropathy Caused by Unusual Polymerization of IgA1 with Aberrant N-Glycosylation in a Patient with Monoclonal Immunoglobulin Deposition Disease

**DOI:** 10.1371/journal.pone.0091079

**Published:** 2014-03-20

**Authors:** Yoshiki Narimatsu, Atsushi Kuno, Hiromi Ito, Hiroyuki Kaji, Syuzo Kaneko, Joichi Usui, Kunihiro Yamagata, Hisashi Narimatsu

**Affiliations:** 1 Research Center for Medical Glycoscience (RCMG), National Institute of Advanced Industrial Science and Technology (AIST), Tsukuba, Ibaraki, Japan; 2 Department of Biochemistry, Life Sciences and Social Medicine, School of Medicine, Fukushima Medical University, Fukushima, Japan; 3 Department of Nephrology, Graduate School of Comprehensive Human Sciences, University of Tsukuba, Tsukuba, Ibaraki, Japan; Institut national de la santé et de la recherche médicale (INSERM), France

## Abstract

Immunoglobulin A nephropathy (IgAN) is a form of chronic glomerulonephritis characterized by the deposition of IgA immune complexes in the glomerular region. The cause of IgAN is unknown, but multiple mechanisms have been suggested. We previously reported a rare case of mesangioproliferative glomerulonephritis in a patient with monoclonal immunoglobulin deposition disease associated with monoclonal IgA1. In this study, we performed the detailed analyses of serum IgA1 from this patient in comparison with those from patients with mIgA plasma cell disorder without renal involvement and healthy volunteers. We found unusual polymerization of IgA1 with additional *N*-glycosylation distinctive in this patient, which was different from known etiologies. Glycan profiling of IgA1 by the lectin microarray revealed an intense signal for *Wisteria floribunda* agglutinin (WFA). This signal was reduced by disrupting the native conformation of IgA1, suggesting that the distinct glycan profile was reflecting the conformational alteration of IgA1, including the glycan conformation detected as additional *N*-glycans on both the heavy and light chains. This unusually polymerized state of IgA1 would cause an increase of the binding avidity for lectins. WFA specifically recognized highly polymerized and glycosylated IgA1. Our results of analysis in the rare case of a patient with monoclonal immunoglobulin deposition disease suggest that the formation of unusually polymerized IgA1 is caused by divergent mechanisms including multiple structural alterations of glycans, which contributes to IgA1 deposition and mesangium proliferation.

## Introduction

Glycosylation of IgA plays a crucial role in its conformational stability and biological activity, and often contributes to disease pathogenesis. Abnormalities of IgA1 glycosylation are implicated in several diseases including IgA nephropathy (IgAN) [1]–[3]. IgAN is the most common primary glomerulonephritis involving selective mesangial deposition of IgA1-containing immune complexes [4], [5]. Many studies have focused on the *O*-glycan structures of IgA, and aberrant galactosylation has been detected in the serum of IgAN patients by lectin ELISA or detecting autoantibodies against exposed *N*-acetylgalactosamine (GalNAc) residues in the *O*-glycan of IgA1 [4]–[7]. Although aberrant *O*-glycosylation of serum IgA1 in IgAN has been studied using various lectins that bind to GalNAc residues, few studies have attempted to purify or enrich these abnormal IgA1 molecules. Moreover, involvement of these glycan alterations in IgAN pathogenesis is unclear, and whether such aberrant IgA1 is accumulated in the mesangium has not been elucidated yet.

We previously reported a rare case of monoclonal immunoglobulin deposition disease associated with monoclonal IgA (mIgA-MIDD) [8]. MIDD is a severe complication of immunoproliferative disorders characterized by monoclonal expansion of the plasma cell population of particular B cells. Interestingly, the pathological findings in this case included proliferative glomerulonephritis with mIgA deposition predominantly in the mesangium. We conducted a mass spectrometry-based analysis to identify and compare the glycan structures of serum IgA1 in this patient, disease control patients with monoclonal IgA plasma cell disorder (MPCD) accompanied by multiple myeloma and monoclonal gammopathy of undetermined significance without renal involvement, and healthy volunteers (HV). We observed a slight increase of IgA1 with core fucosylation and sialylation of *N*-glycans in mIgA-MIDD and similar *O*-glycan profiles among the subjects. However, we could not find evidence for the alterations of these *N*-glycans that caused mesangial IgA1 deposition, because core fucosylation and sialylation of *N*-glycans are often found in normal IgA1. This was probably because we had focused on the primary structures of glycan monomers. However, the secondary structure including the number and position of glycosylations is also important for carbohydrate–protein interactions [9], [10]. Therefore, the secondary and tertiary structures should be investigated by approaches without the release of glycans from glycoproteins. IgA1 is often expressed as multimers such as dimeric or highly polymeric forms [11], emphasizing the importance of analyzing the native properties including conformational and polymerization characteristics.

In this study, we identified distinctive characteristics in the native conformation of IgA1, and detected unusual polymerization of IgA1 in mIgA-MIDD only, which we believed to have caused the IgA1 deposition and mesangium proliferation in this patient.

## Materials and Methods

### Serum samples

Serum samples were collected from three HVs and two MPCD patients and one mIgA-MIDD patient [8] after obtaining written informed consent. All experiments involving human samples were approved by the ethics committees of the University of Tsukuba and AIST, and were conducted in accordance with the Declaration of Helsinki.

### Purification of IgA1

Serum albumin was removed by pretreatment with Cibacron Blue 3GA Agarose (Sigma, St. Louis, MO). A prepacked Cibacron Blue column (1 mL) was washed with a mixture of 1 M NaCl and 0.1 M borate (10 mL, pH 9.8) followed by 10 mL distilled water, and then equilibrated with 10 mM Tris-HCl (10 mL, pH 7.4). The flow-through fraction and wash were collected and combined as the IgA1 fraction, and then purified by affinity chromatography using an anti-human IgA1 monoclonal antibody (anti-IgA1 mAb 7303B, Institute of Immunology, Tokyo, Japan) coupled to cyanogen bromide-activated Sepharose 4FF (1 mg IgG/mL gel; Buckinghamshire, UK). The fraction was loaded onto the anti-IgA1 mAb-coupled Sepharose 4B column (2 mL; GE Healthcare) and washed with 20 mL PBS. The bound IgA1 was eluted with 0.1 M glycine (pH 2.5) and immediately neutralized with 1 M Tris-HCl (pH 8.0). The remaining IgG was removed by mixing the eluate with a protein G bead suspension (Thermo Fisher Scientific, Waltham, MA) and incubating at 4°C overnight. The supernatant was collected, dialyzed against 10 mM ammonium bicarbonate, and then lyophilized.

### SDS-PAGE and western blot analysis

Purified IgA1 was isolated by 10% SDS-PAGE under reducing or nonreducing conditions and then transferred to a PVDF membrane (GE Healthcare). The membrane was blocked with 3% BSA in PBS containing 0.1% Tween 20 (PBS-T), followed by incubation with the anti-IgA1 mAb (1∶1000) and a peroxidase-conjugated anti-mouse IgG (1∶5000, GE Healthcare). For lectin blot analysis, HRP-conjugated WFA (1∶1000) or jack bean lectin concanavalin A (ConA, 1∶4000) were used. Cross-reacting bands were detected using Konica immunostaining kit (Konica, Tokyo, Japan) for anti-IgA1 mAb and ConA and Western Lightning Chemiluminesce nce Plus (Perkin-Elmer, Boston, MA) for WFA.

### Antibody-overlay lectin microarray

Antibody-overlay lectin microarray was performed as described previously [12], [13]. Purified IgA1 was diluted to 80 µL with PBS containing 0.1% Triton X-100 (PBS-Tx) and applied to the lectin microarray. After incubation at 20°C for 18 h, 1 µg human serum polyclonal IgG was added to the array followed by 1 h of incubation. The reaction solution was discarded, and the array was washed with PBS-Tx. A biotinylated anti-IgA1 mAb (400 ng) diluted in 80 µL PBS-Tx was applied to the array followed by incubation at 20°C for 1 h. The array was washed with PBS-Tx and then incubated at 20°C for 30 min with 400 ng Cy3-labeled streptavidin (GE Healthcare) diluted in 80 µL PBS-Tx. The array was rinsed with PBS-Tx and scanned by an evanescent-field fluorescence scanner (GlycoStation; Moritex, Tokyo, Japan). All data were analyzed with Array Pro analyzer (ver. 4.5). The net intensity was calculated by subtracting the mean background value from the mean signal intensity of three spots per lectin. The obtained signal intensities are available as Table S1, S2,S3 as well as at figshare.com (10.6084/m9.figshare.894402).

### Tryptic digestion of IgA1

Purified IgA1 (500 µg) was reduced at 37°C in the presence of 0.1 M Tris-HCl (pH 7.4) and 10 mM DTT, followed by alkylation with 50 mM iodoacetamide at room temperature for 90 min in the dark. The alkylated sample was then dialyzed against 50 mM ammonium bicarbonate (2.5 L, pH 8.5) three times at 4°C for 48 h. The sample (100 µg) was incubated with trypsin (1 µg, Wako Pure Chemical Industries, Osaka, Japan) at 37°C for 18 h. To desalt the tryptic peptides, the sample was applied to a reverse-phase column (Oasis HLB cartridge, 10 mg/mL; Waters, Milford, MA) preconditioned with methanol (3 mL) and distilled water (3 mL). The column was washed with distilled water (3 mL), and the tryptic peptides were eluted with 1 mL of 80% acetonitrile and then lyophilized.

### Lectin microarray analysis of tryptic peptides

Tryptic peptides (1 µg) were labeled with Cy3-succinimidyl ester (10 µg, GE Healthcare) at room temperature for 1 h in the dark. To block the functional groups of the fluorescent reagent, a probe buffer (500 mM glycine in TBS containing 1% Triton X-100) was added to the labeled peptides followed by incubation for 2 h at room temperature in the dark. The Cy3-labeled glycopeptide solution (80 µL, 10 µg/mL) was applied to the lectin microarray. The array was incubated at 20°C for 12 h, washed with the probe buffer, and then scanned by the GlycoStation.

### Sequential exoglycosidase treatment of IgA1

Purified IgA1 (5 µg) was digested sequentially with exoglycosidases at 37°C under the following conditions: neuraminidase from *Arthrobacter ureafaciens* (Nacalai Tesque, Kyoto, Japan), 5 mU in 25 µL of 80 mM sodium acetate buffer (pH 5.0) for 18 h; β-galactosidase from bovine testes (Sigma), 2.2 mU in 5 µL of 80 mM sodium acetate buffer (pH 5.0) overnight; β1,4-galactosidase from *Streptococcus pneumoniae* (Calbiochem, La Jolla, CA), 3 mU in 5 µL of 80 mM sodium acetate buffer (pH 5.0) overnight.

### ELISA using various lectins

ELISA plates (MaxiSorp 96-well immunoplates; Thermo Fisher Scientific) were coated with the F(ab′)_2_ fragment of 5 µg/mL goat anti-human serum IgA (50 µL/well, Jackson ImmunoResearch Labs, West Grove, PA) in PBS and then incubated at room temperature for 1 h. The plates were then blocked with 1% BSA in PBS and incubated with 50 µL deglycosylated IgA1 (5 µg/mL) at 4°C overnight. Subsequently, the plates were washed with PBS-T and incubated with each biotinylated lectin: *Helix pomatia* agglutinin (HPA, 5 µg/mL; EY Laboratories, San Mateo, CA), *Vicia villosa* agglutinin (VVA, 5 µg/mL; Vector Laboratories), peanut agglutinin (PNA, 2 µg/mL; Vector Laboratories, Burlingame, CA), and WFA (5 µg/mL; Vector Laboratories) at room temperature for 2 h. After washing, the plates were incubated with HRP-conjugated streptavidin at room temperature for 1 h. A 3,3′,5,5′-tetramethylbenzidine substrate solution (50 µL/well, Thermo Fisher Scientific) was added to the plates followed by incubation at room temperature until signal development. The reaction was stopped with 2 M sulfuric acid (50 µL), and the optical density was measured at 450 nm with a Wallac 1420 ARVO SX Multilabel Counter (Perkin Elmer).

### De-N-glycosylation of purified IgA1 by peptide N-glycosidase F

Purified IgA1 (4 µg) was pretreated with 0.5% SDS and 0.2 M 2-mercaptoethanol (2-ME) at 98°C for 5 min and then incubated with peptide *N*-glycosidase F (PNGase F, Takara Bio, Shiga, Japan) in the presence of 1% Nonidet P-40 (Sigma). For each partial digestion, pretreated IgA1 (2.5 µg) was treated with 0.1 mU PNGase F in PBS (pH 8.1) at 37°C. Complete de-*N*-glycosylation was performed with 1 mU PNGase F at 37°C overnight.

### SDS-agarose gel electrophoresis

Analysis was performed as described previously [12]. Purified IgA (5 µg) was electrophoresed in TBS buffer at 100 V for 40 min under nonreducing conditions and then transferred to a PVDF membrane. The membrane was blocked with 3% BSA in PBS-T and then incubated with peroxidase-conjugated anti-human IgA1 (1∶5000; Dako North America, Carpinteria, CA). After washing, cross-reacting bands were detected using Western Blue detection reagent (Promega, Madison, WI).

## Results

### Comparative glycoprofiling of whole IgA1 molecules using a lectin microarray

**Figure 1:**
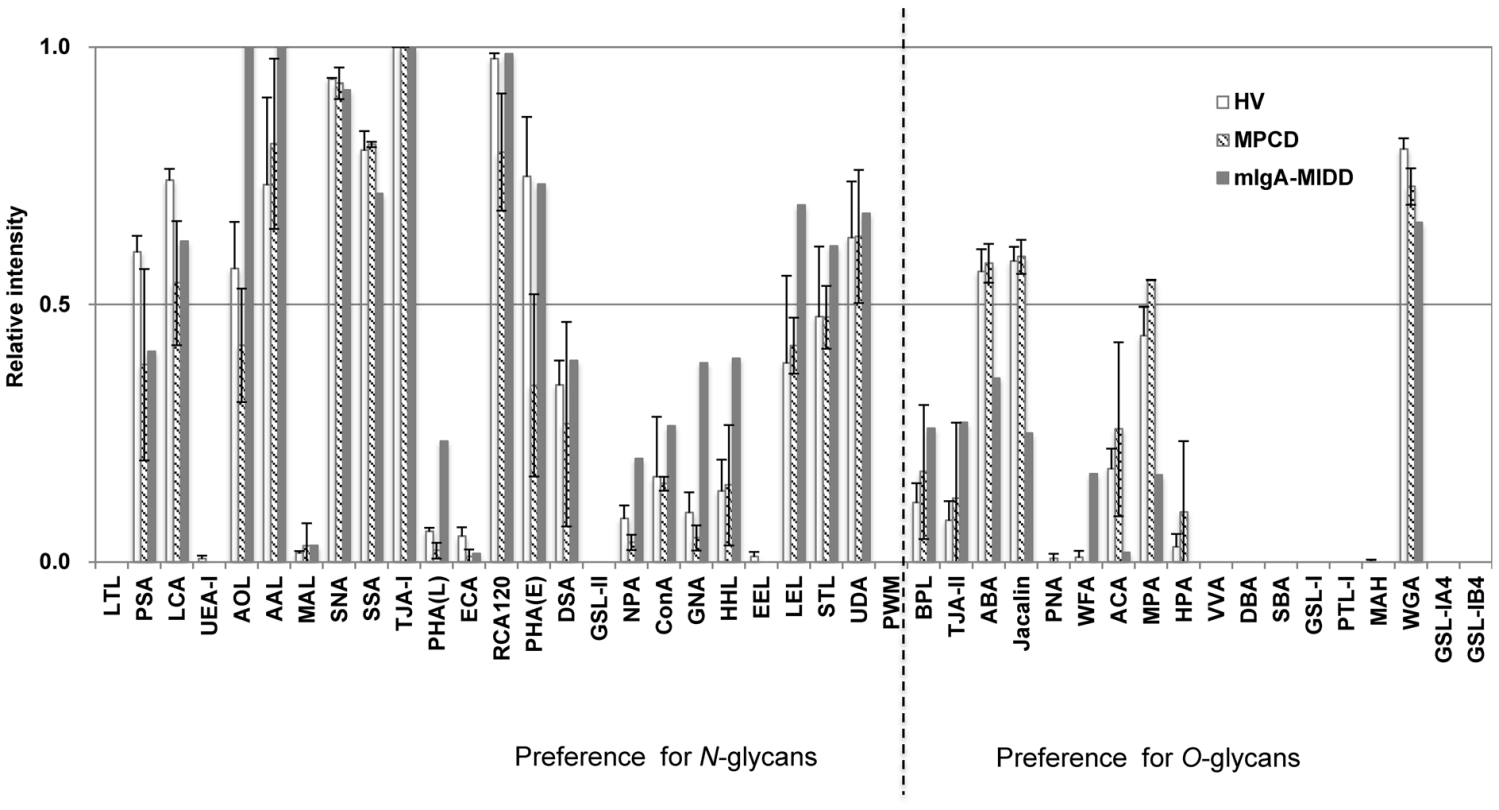
Differential glycan profiles of purified IgA1. IgA1 purified from sera of three HVs, two MPCD patients, and one mIgA-MIDD patient was subjected to lectin microarray. IgA1-binding signals on the lectin microarray were detected with a biotinylated anti-IgA1 mAb. The relative intensity of each lectin was normalized to the maximum fluorescence intensity. mIgA-MIDD, monoclonal immunoglobulin deposition disease associated with monoclonal IgA; MPCD, monoclonal IgA plasma cell disorder; HV, healthy volunteers.

We compared the glycan profiles of serum IgA1 collected from the mIgA-MIDD patient, two MPCD patients, and three HVs. IgA1 was enriched from individual serum by affinity column chromatography with an anti-IgA1 monoclonal antibody and subjected to SDS-PAGE, Western blotting, and the antibody-overlay lectin microarray with an anti-IgA1 mAb detection system. Compared with HVs and MPCDs, the heavy chain of IgA1 from mIgA-MIDD had a slightly increased molecular weight (Figure S1). Signal alterations in some *O*-glycan binders and differences in the signal patterns of several *N*-glycan binders, such as *Hippeastrum hybrid* lectin (HHL), *Galanthus nivalis* agglutinin (GNA), and *Phaseolus vulgaris* leucoagglutinin (PHA-L) [13], were observed in mIgA-MIDD (Figure 1). A significant difference in the signal of *Wisteria floribunda* agglutinin (WFA) was also observed in mIgA-MIDD, which may not have been caused solely by *O*-glycans [14].

### Comparative glycoprofiling of trypsin-digested IgA1

**Figure 2:**
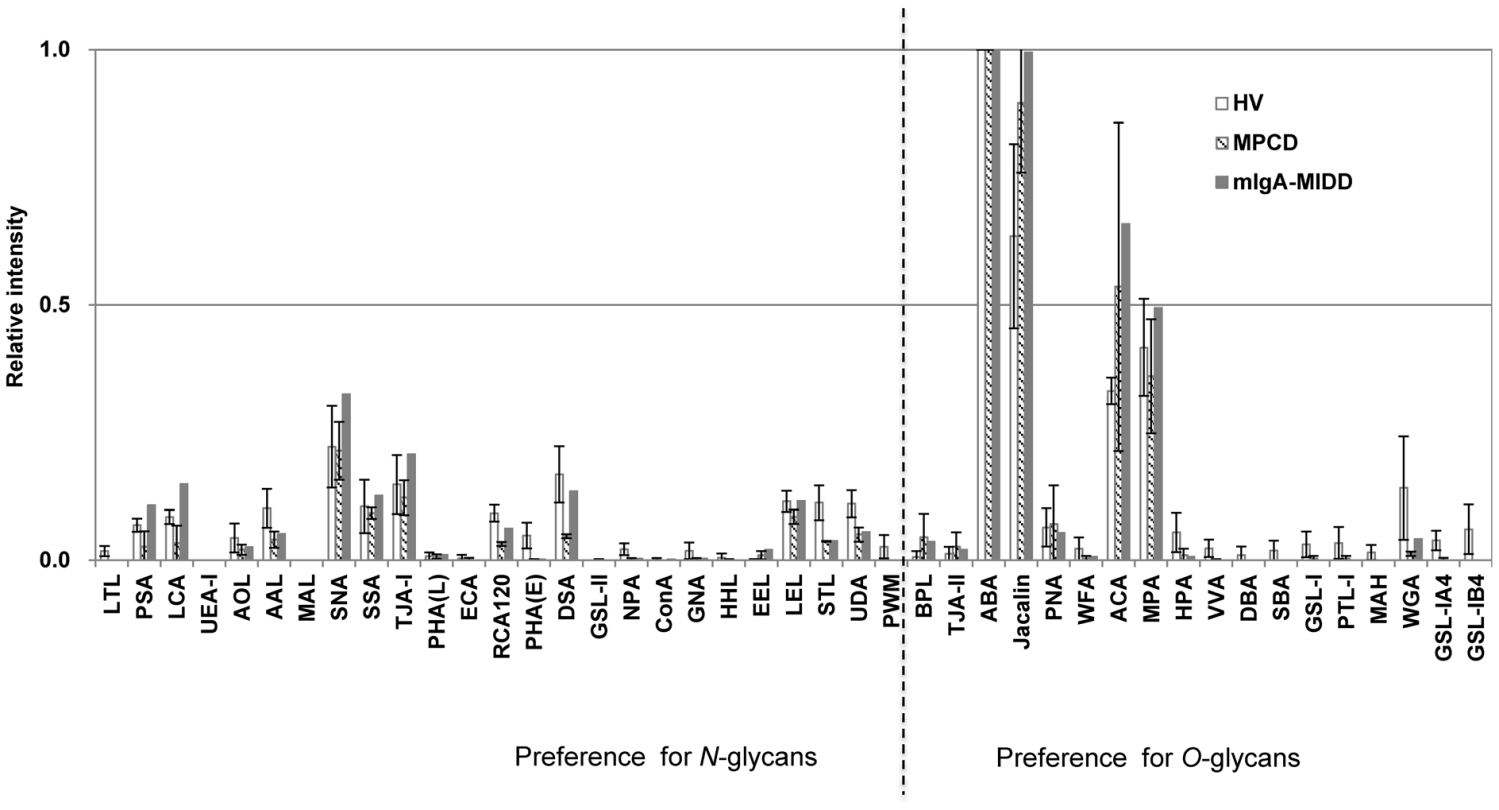
Differential glycan profiles of purified IgA1 after trypsin digestion. IgA1 purified from sera of three HVs, two MPCD patients, and one mIgA-MIDD patient was incubated with trypsin. Each tryptic digest was labeled with Cy3-SE and subjected to the lectin microarray. The relative intensities of lectins were normalized to the maximum fluorescence intensity.

Tryptic digests of serum IgA1 contain one *O*-glycopeptide and two *N*-glycopeptides, each carrying five *O*-glycans and one *N*-glycan, respectively [15], [16]. Trypsin digestion exposes *O*-glycans, and thus the resultant profiles of samples should be limited mostly to *O*-glycan structures. We performed lectin microarray analysis of IgA1-derived tryptic glycopeptides using a direct fluorescence labeling method [13], [17]. As shown in Figure 2, in mIgA-MIDD, the lectin signals that prefer *O*-glycans (e.g., *Agaricus bisporus* agglutinin [ABA], Jacalin, PNA, and *Amaranthus caudatus* agglutinin [ACA]) were not largely affected, whereas the trend of signals became comparable to those of HVs and MPCDs. Interestingly, the strong WFA signal in whole IgA1 from mIgA-MIDD was diminished by tryptic digestion, but the reactivity of other *O*-glycan binders was similar among the subjects. These results indicated that the signal enhancement of WFA observed in IgA1 of mIgA-MIDD was based on the interactions of *N*-glycans but not *O*-glycans.

**Figure 3:**
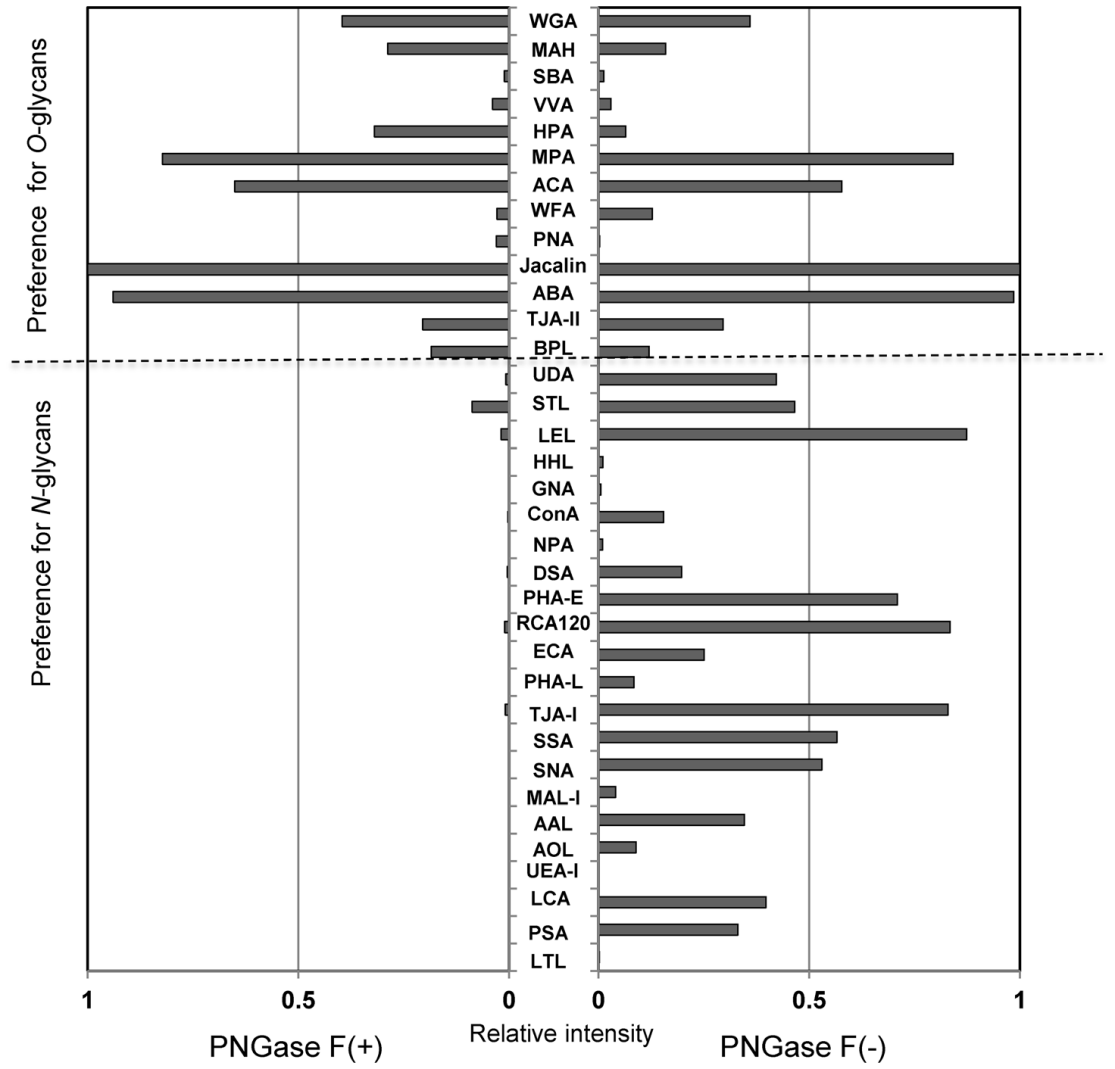
Differential glycan profiles of PNGase F-treated IgA1 of mIgA-MIDD. IgA1 purified from mIgA-MIDD serum was incubated with (left) or without (right) PNGase F. After digestion, the reaction mixture was labeled with Cy3-SE and subjected to the lectin microarray. The relative intensity of each lectin was normalized to the maximum fluorescence intensity. PNGase F, peptide *N*-glycosidase F.

To confirm the binding preference of WFA to *N*-glycans, IgA1 was de-*N*-glycosylated by PNGase F. As shown in Figure 3, signals of all *N*-glycan binders were diminished after digestion, while the *O*-glycan binders still showed equal or much stronger binding than those without treatment. The WFA signal was also reduced by de-*N*-glycosylation, indicating that the enhanced WFA binding in whole IgA1 was associated with the change of *N*-glycans.

### WFA binds to IgA1 macromolecules

**Figure 4:**
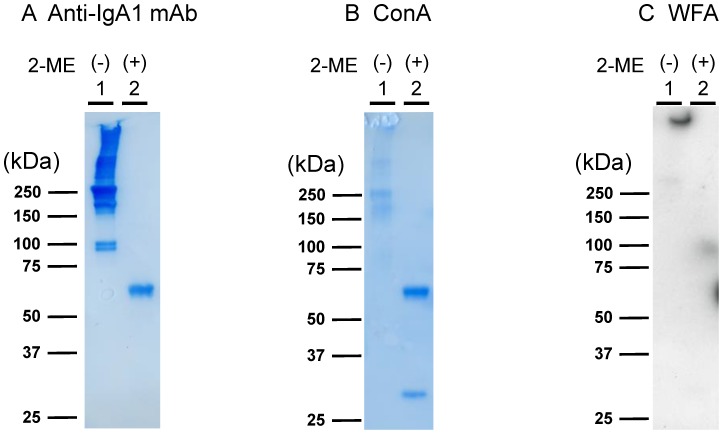
Lectin blot analysis of IgA1 purified from mIgA-MIDD serum under reducing and non-reducing conditions. IgA1 purified from mIgA-MIDD serum was pretreated in SDS buffer with or without 2-ME, separated by SDS-PAGE under non-reducing (lane 1) or reducing (lane 2) conditions, and then subjected to western blotting with an anti-IgA1 mAb (A), ConA (B), and WFA (C). Cross-reacting bands were detected using Konica immunostaining kit (Konica, Tokyo, Japan) for anti-IgA1 mAb and ConA and Western Lightning Chemiluminescence Plus (Perkin-Elmer, Boston, MA) for WFA. 2-ME, 2-mercaptoethanol; ConA, jack bean lectin concanavalin A; WFA, *Wisteria floribunda* agglutinin.

Lectin microarray and lectin blot analyses of IgA1 were performed under reducing (with 2-mercaptoethanol treatment, 2-ME(+)) and nonreducing (2-ME(−)) conditions (Figure 4). IgA1 was detected using the anti-IgA1 mAb which binds to IgA1 in any conformational state, and ConA, which binds to all *N*-glycans of IgA1 in any conformational state. The WFA signal was decreased in samples not treated with PNGase F but denatured by 2-ME (Figure 4C). This means there was less IgA1 bound to WFA under reducing conditions, and such binding seemed to occur only at the loading pocket of the blot, suggesting aggregation and unusually polymerized state of IgA1, under nonreducing conditions. These results suggest that glycans on intact IgA1 are essential for WFA binding, and that WFA preferentially binds to polymerized IgA1 macromolecules.

### Sandwich lectin ELISA analysis of sequentially deglycosylated IgA1

WFA preferably binds to GalNAc residues [18], thus the IgA1 of mIgA-MIDD was speculated to contain *N,N*′-diacetyllactosamine (GalNAcβ1,4GlcNAc, LacdiNAc) in its *N*-glycans. Although the enhanced WFA signal was observed in the lectin microarray, such WFA ligands were undetectable in our previous MS-based structural analysis [8]. Taken together, these results suggest that WFA binds to a different glycan epitope from those reported previously.

**Figure 5:**
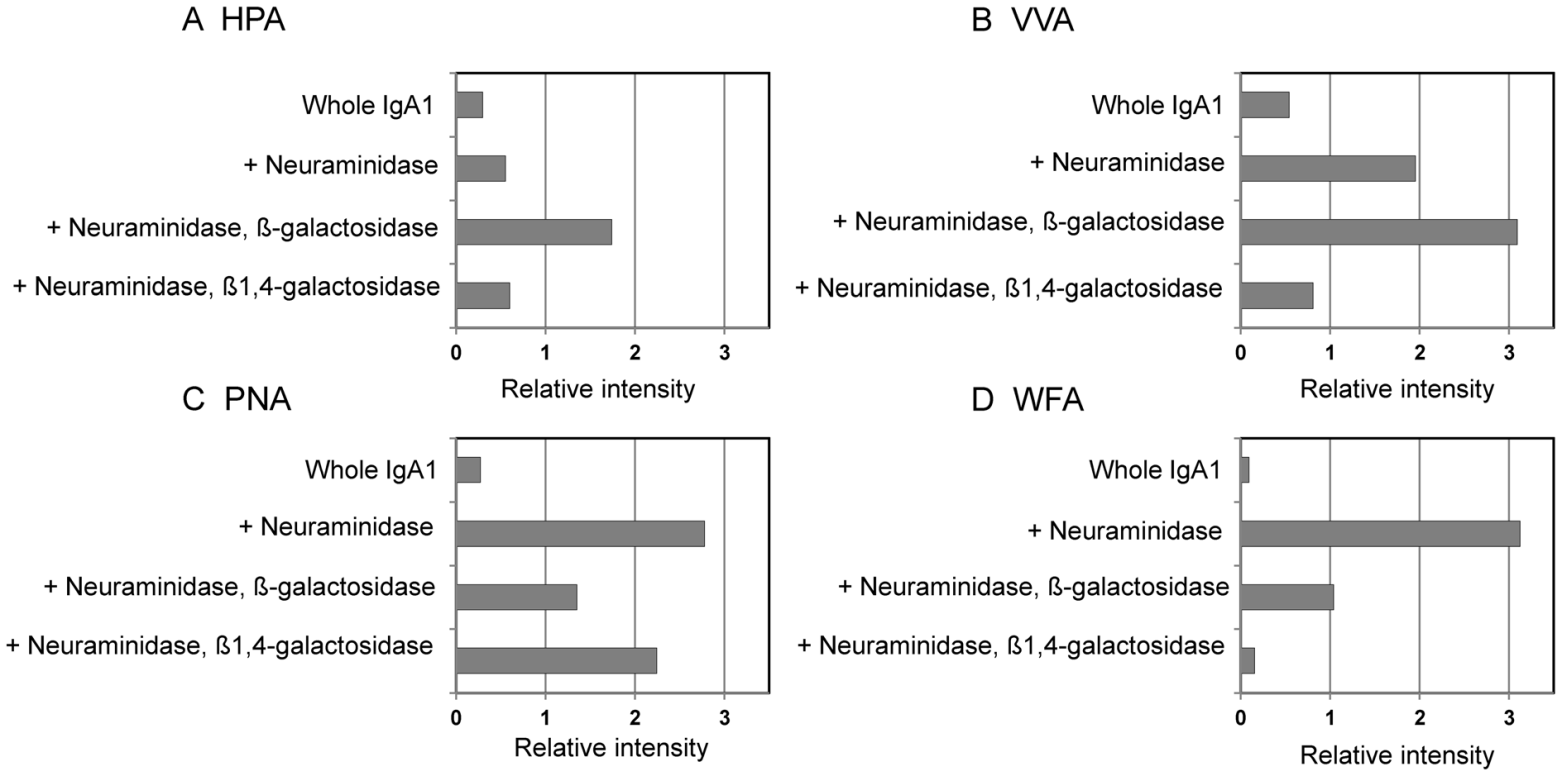
Sandwich lectin ELISA of sequential deglycosylated IgA1 in mIgA-MIDD. IgA1 purified from mIgA-MIDD serum was digested with neuraminidase, and then β-galactosidase or β1,4-galactosidase. Digested and undigested samples were subjected to a sandwich lectin ELISA with HPA (A), VVA (B), PNA (C), and WFA (D) as described in the Methods. The relative intensity of each lectin was normalized to the IgA1 concentration. HPA, *Helix pomatia* agglutinin; VVA, *Vicia villosa* lectin; PNA, peanut agglutinin.

The binding specificities of WFA and other *O*-glycan binders were compared by a lectin ELISA on sequentially deglycosylated IgA1 against HPA, VVA, PNA, and WFA. HPA and VVA bind preferentially to the Tn antigen and terminal GalNAc residues. As expected, the highest binding avidity was observed for HPA and VVA upon sequential digestion with neuraminidase and β-galactosidase, in which the GalNAc residues attached on an *O*-glycan in the hinge region of IgA1 were exposed (Figure 5A and B). This *O*-glycan possesses a Tn antigen, a core-1 structure (Galβ1-3GalNAcα1-Thr/Ser). Thus, it was not exposed by β1,4-galactosidase treatment. PNA specifically recognized Galβ1,3-GalNAc residues (T antigen) and bound strongly to neuraminidase-treated IgA1 with exposed β1,3-Gal residues (Figure 5C). For WFA, the binding avidity was increased by neuraminidase treatment. Interestingly, WFA binding to neuraminidase-treated IgA1 was reduced markedly by sequential treatment with β1,4-galactosidase (Figure 5D). These results strongly suggest that WFA binds to an unknown epitope of *N*-glycans containing the LacNAc unit (Galβ1-4GlcNAc-R).

### Additional N-glycosylation on heavy and light chains of mIgA-MIDD

Multivalency of glycosylation is the most important factor for carbohydrate–protein interactions. Our results also indicated that the multivalent lectin–carbohydrate interaction contributes to WFA binding to IgA1 in mIgA-MIDD. There are several ways for multivalent glycans to undergo more lectin–carbohydrate interactions. First, alterations of glycan structures, such as increasing the multiantennary glycan structures, can enhance the binding avidity for lectins. Second, an increase in the number of glycosylation sites leads to high avidity in the multiple interactions, as observed in clustered mucin *O*-glycosylation. Third, in macromolecules and polymerized glycoproteins, clustered glycans are often present depending on the conformational properties, which enhance lectin–carbohydrate interactions.

**Figure 6:**
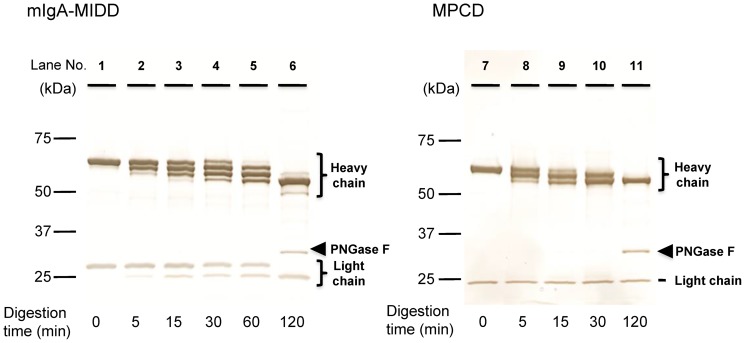
Time course of PNGase F treatment of IgA1. IgA1 purified from the sera of the mIgA-MIDD patient (A) and an MPCD patient (B) was digested partially with PNGase F for the indicated times (lanes 1–5, 7–10). Complete digestion of *N*-glycans was performed by incubation with PNGase F for 120 min (lanes 6, 11). Arrowheads indicate the position of PNGase F.

We investigated the number of *N*-glycans attached to IgA1 of mIgA-MIDD. Purified IgA1 was digested partially or completely with PNGase F and then separated by SDS-PAGE. Before PNGase F treatment, the heavy and light chains of IgA1 purified from mIgA-MIDD and MPCD patients showed 65 and 30 kDa bands (Figure 6, lane 1), and 60 and 25 kDa bands (lane 7), respectively. Partial PNGase F digestion of IgA1 from mIgA-MIDD and MPCD patients showed four bands (lane 4) and three bands (lane 9), respectively. Complete PNGase F digestion of IgA1 caused the bands to converge into single bands (lanes 6 and 11). These results indicate that IgA1 of MPCD possesses two *N*-glycans in the heavy chain, which is consistent with normal IgA1 having two *N*-glycans in the constant region [16]. In contrast, IgA1 of mIgA-MIDD possesses a third *N*-glycan in addition to the same two *N*-glycans found in MPCD and normal IgA1. Moreover, similar to normal IgA1, the band with the higher molecular mass of the light chain in IgA1 of mIgA-MIDD was shifted after complete digestion (lanes 1 and 6), indicating that the light chain also possesses an *N*-glycan. These results suggest that IgA1 of mIgA-MIDD possesses an aberrant *N*-glycan on both heavy and light chains, resulting in eight *N*-glycans in monomeric IgA1; three on the heavy chain and one on the light chain. This high *N*-glycosylation may result in a remarkably aberrant conformation of IgA1.

### Formation of a high molecular complex of IgA1 in mIgA-MIDD

Human serum IgA1 has a distinct polymerization state including monomeric, dimeric, and highly polymeric forms, and most serum IgA1 exists normally in the monomeric form (90–95% of total serum IgA1) [11].

**Figure 7:**
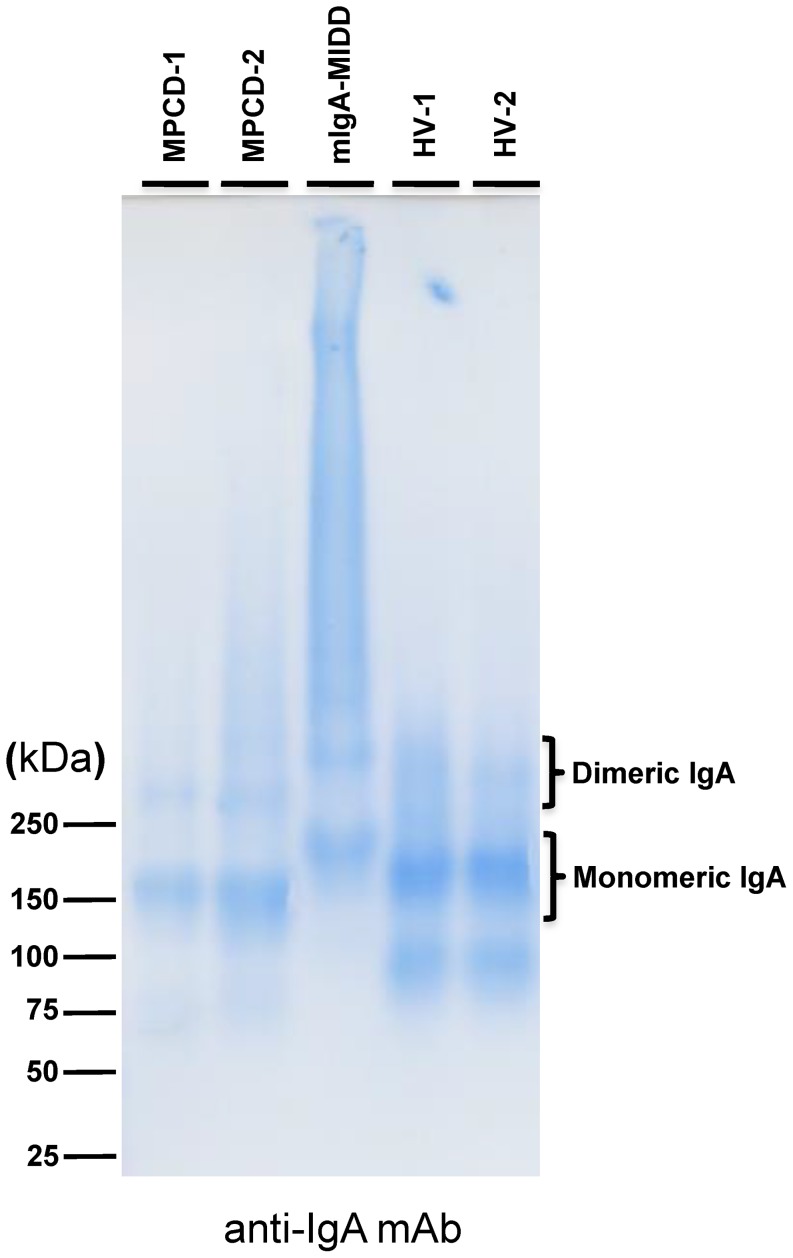
SDS-agarose gel electrophoresis of purified IgA1. IgA1 purified from sera of two HV, two MPCD patients, and an mIgA-MIDD patient was incubated in SDS buffer and then subjected to agarose gel electrophoresis analysis.

To investigate the polymerization state of whole IgA1 in mIgA-MIDD, SDS-PAGE was performed under reducing and nonreducing conditions (Figure S2). In the denatured state, IgA1 of mIgA-MIDD had a similar molecular mass compared with that of IgA1 from MPCD patients and HVs, although an increase in the number of *N*-glycans resulted in slower migration of the heavy chain (Figure 4, Figure S2). Because a strong band with a high molecular mass was found in the denaturing SDS-PAGE without 2-ME treatment in mIgA-MIDD, we performed SDS-agarose gel electrophoresis under nonreducing conditions. As shown in Figure 7, IgA1 of MPCD patients and HVs appeared mainly in the monomeric form based on its molecular mass, whereas IgA1 of mIgA-MIDD appeared predominantly in the high molecular mass form, i.e., the unusually polymerized form (Figure 7, lane 3). Thus, the unusually polymerized IgA1 was detected only by SDS-agarose gel electrophoresis under nonreducing conditions, suggesting that the avidity of WFA for *N*-glycans was increased markedly by the clustered *N*-glycans on polymerized IgA1 in mIgA-MIDD.

## Discussion

To explore the distinctive glycans of monoclonal IgA1 in mIgA-MIDD different from the IgA1 of MPCD patients and HVs, we used a lectin microarray to focus on comparative glycan profiling and the structural characteristics of the native conformation of IgA1. Lectin microarrays detect interactions between lectins and glycans. The specificity and avidity of lectin–carbohydrate interactions are influenced by many factors such as structures, orientations, and density of ligands. Formation of a multivalent glycan complex can substantially affect the overall avidity of the interactions and selectivity of recognition [10], [19], [20]. In this context, a lectin microarray is a useful glycoanalytical tool that can also detect multivalency effects.

In this study, we identified unusual binding of WFA to the terminal β1,4-linked galactose residues, but not the LacdiNAc residue, on *N*-glycans (Figure 5). Such binding seemed to be involved only in unusually polymerized IgA1 and not the monomeric, dimeric, or denatured forms of IgA1 (Figure 4). Thus, it is reasonable to assume that the binding ability to weak ligands of WFA, as that of β1,4-linked galactose, was increased to induce lectin–carbohydrate interactions by multivalency effects.

The alterations in glycan multivalency and the physical properties of IgA1 were found only by the glycoprotein assay in combination with heat, tryptic digestion, and PNGase F pretreatment, which disrupted the native conformation of IgA1. The glycoprotein analysis based on the lectinology, including the lectin microarray and lectin-based immunoassay, can be applied efficiently to glycosylation profiling without releasing glycans. Thus, the profiling data of lectin analyses significantly reflect the physical properties of glycoproteins. Lectin analyses with several pretreatment approaches are useful to identify glycoalterations involving the physical properties and structures of glycoproteins.

Glycosylation has a profound effect on the tertiary structure of glycoproteins. Several reports have shown that *O*-glycosylation of serine or threonine significantly affects the peptide backbone conformation [15], [21], [22]. Similarly, *N*-glycosylation also influences protein structures [23]–[25] and decreases the conformational mobility of the peptide backbone at glycosylation sites [26]–[28]. Several in vitro studies showed that attempts to add *N*-glycans result in unpredictable thermodynamic consequences and destabilization [29]–[31]. Another study showed that mutant human IgA1 lacking *N*-glycosylation sites tends to form large polymers [32]. Although there might be a possibility of aggregation or polymerization attributable to the purification process, our results indicated that IgA1 from mIgA-MIDD tends to aggregate more easily than those of HVs or MPCDs. Addition of an *N*-glycan might affect the overall tertiary structure or surface properties of the protein. These changes are attributable to the increase or decrease caused by one *N*-glycosylation. Additional aberrant *N*-glycosylation is found frequently in variable regions of the Ig light chain and in about 15% of the circulating light chain-containing *N*-glycosylation in multiple myeloma patients [33]–[35]. Although unusually polymerized states are less common among these aberrantly *N*-glycosylated Igs, several studies show that an additional *N*-glycosylation site on the light chain results in tissue deposition of the monoclonal light chains and insoluble fibrillization in patients with primary amyloidosis and light chain deposition disease [36], [37]. Normal IgA1 possesses two *N*-glycosylation sites on its Fc region of the heavy chain [16], [38], [39]. In mIgA-MIDD, we identified an additional *N-*glycosylation site on each of the heavy and light chains. The additional aberrant *N*-glycosylation may influence the conformation and overall stability of the protein structure, leading to polymerized IgA1 with a high molecular mass.

Unusual polymeric IgA1 with aberrant *N*-glycosylation has not been reported in a patient with renal involvement. Selective IgA1 deposition in the renal glomerular mesangium is a typical characteristic of IgAN. Polymeric IgA1 plays a critical role in mesangial deposition and proliferation [40], [41]. Unusually polymerized IgA1 may explain IgA1 deposition in the glomerular mesangium in mIgA-MIDD. Although the importance of polymerization is generally accepted, opinions vary regarding the polymerization mechanisms in IgAN. There are several reports on the induction of polymeric IgA1. Some studies showed that the alteration of *O*-glycan structures results in the autoimmune reaction (formation of immune complexes) in the serum from IgAN patients.[4]–[6], In our study, we could not confirm the presence of immune complex from our experiments. Further studies are required to elucidate the mechanism of the unusual polymeric IgA1 formation. There have been only a few studies on aberrant *N*-glycosylation in human IgAN [42], [43], but alteration of *N*-glycan structures has been reported in mouse IgAN that spontaneously develops glomerulonephritis resembling human IgAN with high serum IgA levels and an increase of polymeric IgA [44]. Taken together, our findings suggest that the aberrant additional *N*-glycosylation of IgA1 also participates in polymerization of IgA1 in human IgAN. IgA1 deposition and mesangial proliferation may be caused by divergent pathogenic mechanisms involving changes of both *O*- and *N*-glycosylation.

We conclude that aberrant additional *N*-glycosylation on both light and heavy chains induces conformational changes of IgA1, resulting in polymerization and glomerular deposition in this rare case of the mIgA-MIDD patient. Further studies are needed to identify the peptide sequence of the additional two *N*-glycosylation sites and the detailed polymerization mechanism. Qualitative and quantitative analyses of aberrant IgA1 might allow detection of unusual polymeric IgA1 in the serum from IgAN patients. Detection of unusually polymerized IgA1 may contribute to elucidating the pathogenesis of renal involvement in MIDD and IgAN.

## Supporting Information

Figure S1 **SDS-agarose gel electrophoresis of purified IgA1.** IgA1 purified from serum of HV, MPCD, and mIgA-MIDD was subjected to SDS-PAGE, and then to silver staining (A) and Western blotting with anti-IgA1 mAb (B). Due to the characteristics of plasma cell disorders, B cell monoclonal expansion, the heavy and light chains of MPCDs and mIgA-MIDD show sharp bands with individual variety of the molecular weight of the light chain.
(TIF)

Figure S2 **SDS-polyacrylamide gel electrophoresis of purified IgA1.** IgA1 purified from HV, MPCD, and mIgA-MIDD was boiled in SDS buffer with (A) or without (B) 2-ME and subjected to SDS-PAGE.
(TIF)

Table S1 **Lectin array signal intensities for purified IgA1(Individual data).**
(XLSX)

Table S2 **Lectin array signal intensities for purified IgA1 after trypsin digestion (Individual data).**
(XLSX)

Table S3 **Lectin array signals for PNGase F-treated IgA1 of mIgA-MIDD.**
(XLSX)
