# Supplementary figures and images for: IgA Nephropathy Caused by Unusual Polymerization of IgA1 with Aberrant N-Glycosylation in a Patient with Monoclonal Immunoglobulin Deposition Disease
Source: PLoS One. 2014 Mar 20;9(3):e91079. doi: 10.1371/journal.pone.0091079 (PMC3961232; doi:10.1371/journal.pone.0091079)

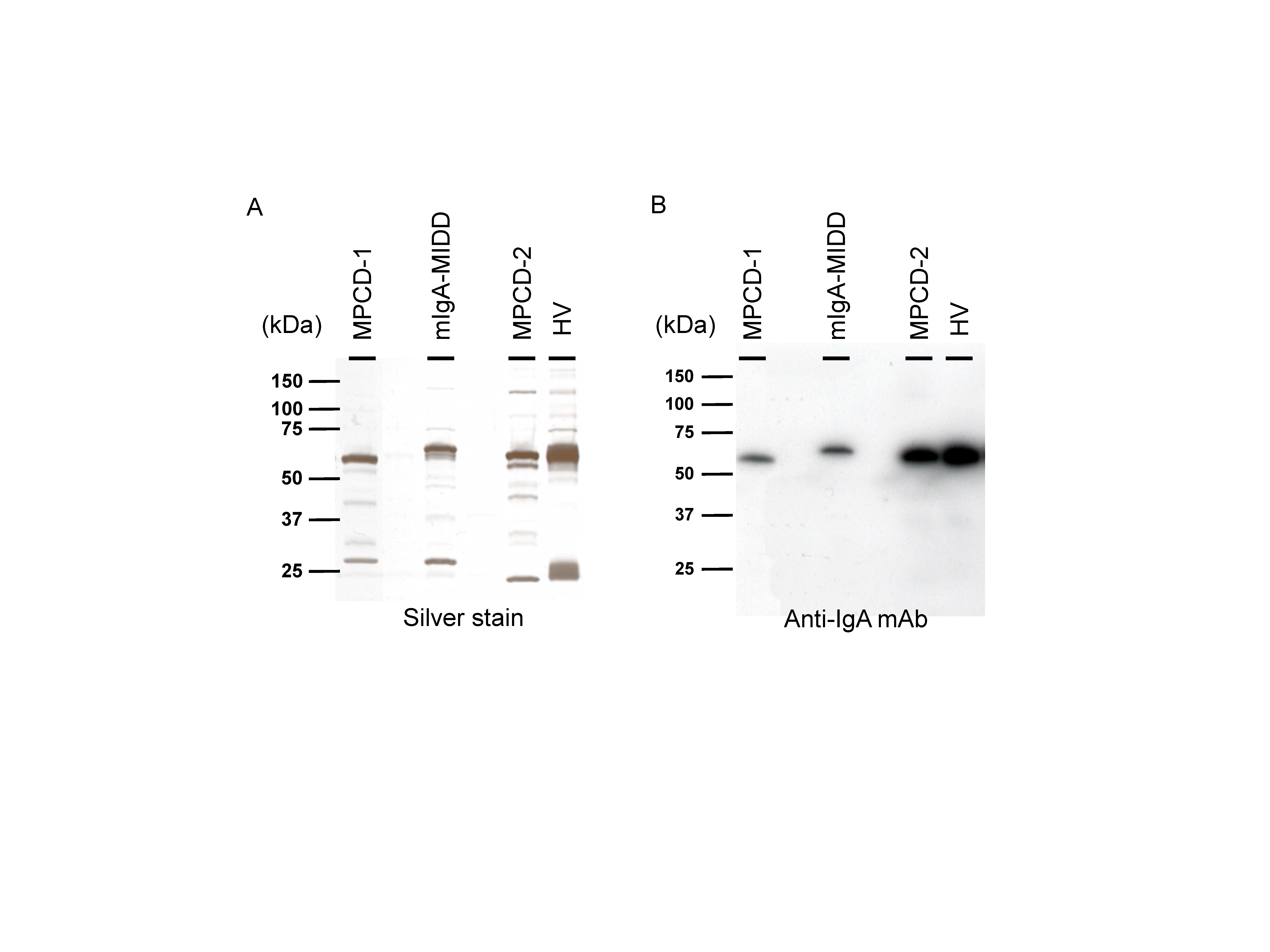

Supplement: Figure S1 — SDS-agarose gel electrophoresis of purified IgA1. IgA1 purified from serum of HV, MPCD, and mIgA-MIDD was subjected to SDS-PAGE, and then to silver staining (A) and Western blotting with anti-IgA1 mAb (B). Due to the characteristics of plasma cell disorders, B cell monoclonal expansion, the heavy and light chains of MPCDs and mIgA-MIDD show sharp bands with individual variety of the molecular weight of the light chain. (TIF) [file pone.0091079.s001.tif]

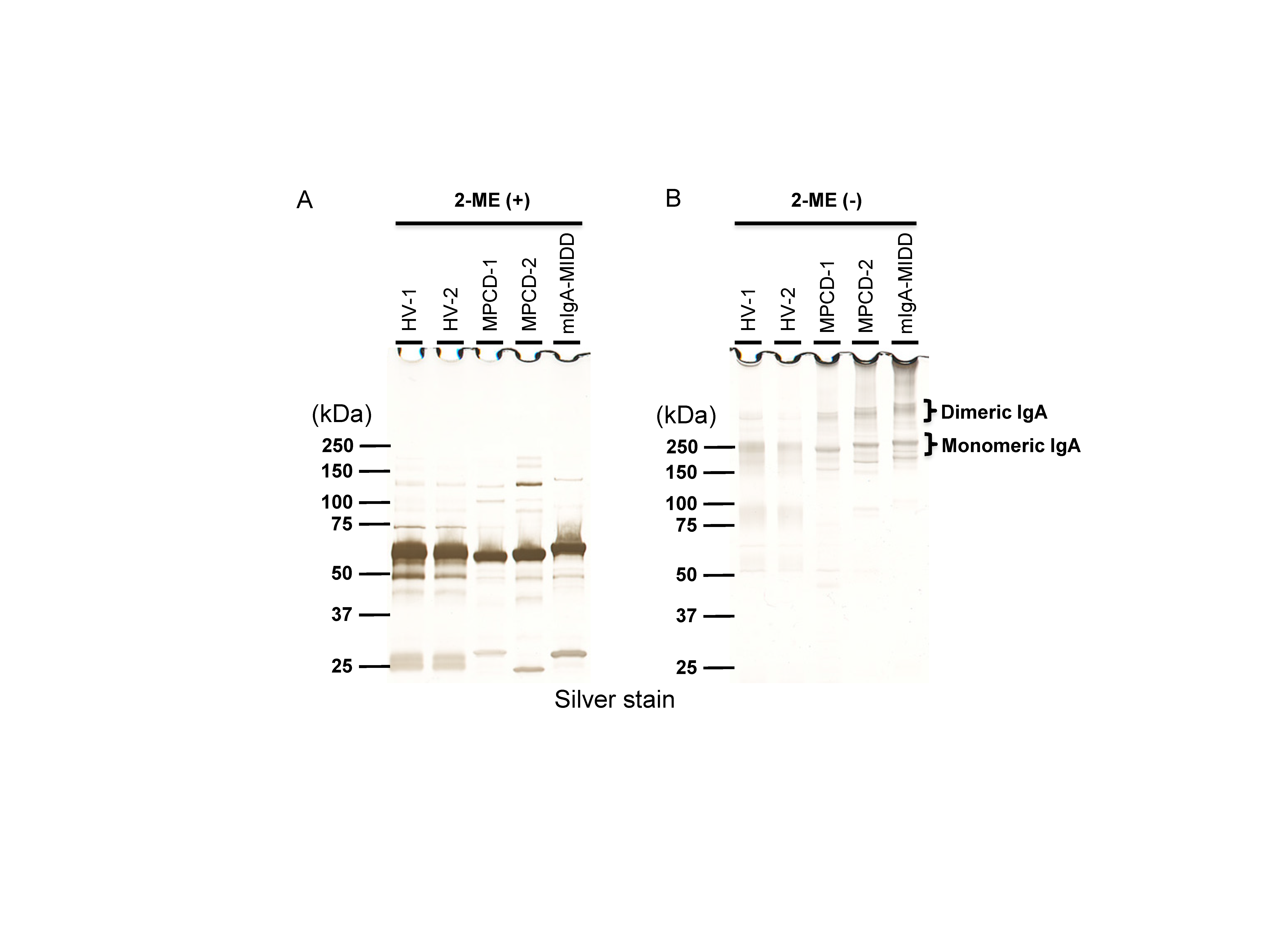

Supplement: Figure S2 — SDS-polyacrylamide gel electrophoresis of purified IgA1. IgA1 purified from HV, MPCD, and mIgA-MIDD was boiled in SDS buffer with (A) or without (B) 2-ME and subjected to SDS-PAGE. (TIF) [file pone.0091079.s002.tif]
